# Prevalence and Risk Factors of Work-Related Upper Extremity Disorders among University Teaching Staff in Ethiopia, 2021: An Institution-Based Cross-Sectional Study

**DOI:** 10.1155/2022/7744879

**Published:** 2022-05-14

**Authors:** Amensisa Hailu Tesfaye, Tesfaye Hambisa Mekonnen, Mekuriaw Alemayehu, Giziew Abere

**Affiliations:** Department of Environmental and Occupational Health and Safety, Institute of Public Health, College of Medicine and Health Sciences, University of Gondar, Gondar, Ethiopia

## Abstract

**Background:**

Work-related upper extremity disorders (WRUEDs) are aches, pains, tension, and discomfort in the neck, shoulders, arms, wrists, hands, and fingers. The situation is escalating in educational sectors due to a lousy working environment intertwined with extracurricular deeds. However, empirical evidence focusing on academicians in higher education society is negligible. The purpose of this study is to examine the prevalence and risk factors of WRUEDs among university teaching staff in Ethiopia.

**Materials and Methods:**

We conducted a cross-sectional study design from March to April 2021. A sample of 607 academicians were recruited using a stratified sampling technique, and a self-administered structured Nordic Musculoskeletal questionnaire was used to assess upper extremity disorders during the past 12 months. The collected data were entered into EpiData version 4.6 and analyzed using STATA version 14 software. The association between dependent and independent variables was computed with a binary logistic regression. The association was ascertained using an adjusted odds ratio (AOR) with a 95% confidence interval (CI) at a *p* value of <0.05.

**Results:**

A total of 607 participants correctly completed the questionnaire (response rate of 95.44%). Age ranges from 21 to 70 with a mean of 32.39 (SD ± 6.80)) years, and the majority (76.28%) of them were males. The prevalence of WRUED during the last 12 months was 59.14% [95% CI (55.1, 63.1)]. There is no significant difference in prevalence between males and females (45.14% versus 14%), respectively; *χ*^2^ = 0.001; *p*=0.974. Working more than 8 hours per day [AOR: 2.37; 95% CI (1.40, 4.00)], not performing physical exercise [AOR: 2.34; 95% CI (1.6, 3.45)], and job dissatisfaction [AOR: 2.50; 95% CI (1.69, 3.68)] were factors significantly increased the risk of experiencing WRUEDs.

**Conclusion:**

This study divulged upper extremity disorder among university teaching staff is pervasive, with more than three-fifth of the academicians were suffering from the condition, and it also indicates that males experienced higher proportions of pain than females. The manifestation of upper extremity disorder was affected by working hours per day, physical activity, and job satisfaction. Optimizing working hours, having a group regular exercise, and proper management of workplace conditions related to job satisfaction are recommended to lessen the condition.

## 1. Background

Work-related upper extremity disorders (WRUEDs) are aches, pains, tension, and discomfort that affect the muscles, tendons, ligaments, nerves, or other soft tissues associated with the neck, shoulders, arms, hands, wrists, and fingers, which can be caused or exacerbated by work and the environment in which it is performed [1, 2]. WRUED is among one of the top 10 work-related conditions and become the most pressing human health issue in the global healthcare system [3]. Besides the impact on patients themselves, the disorders also form a huge economic burden due to costs for sick leave and health care. The financial cost caused by such disorders affects not individual alone but also the organization and the society as a whole [4].

WRUEDs represent more than 67% of all work-related injuries and cost over $110 billion annually for medical expenses, lost wages, and productivity [5]. In affluent countries, the cost of WRUEDs has been estimated between 0.5% and 2% of gross national product (GNP), in addition to its public health effects [6, 7]. For instance, one-third of workers' compensation costs in private industry in the USA is estimated to be caused by WRUED [8], and the direct costs, with compensation, exceed US$ 20 billion in Washington State alone [9]. The Health & Safety Executive, a British institution responsible for the regulation of occupational risks to health, estimated that self-reported WRUED resulted in 4.7 million lost working days in 2003/04 [10]. In poor nations like Ethiopia, where there is a lack of understanding of ergonomics issues, limited training programs, and certification, the impact of WRUEDs is either incalculable or under-reported. As a result, the financial expenditures and healthcare demands associated with upper extremity disorder have skyrocketed, making it a major societal burden [11, 12]. In addition, in the poorest countries, health and safety standards are habitually disregarded, and infrastructure and preventive measures are neglected [13]; as a result, the health effects of WRUEDs have been escalating in these countries.

In recent years, the occurrences of WRUEDs, have been rising rapidly in all working population groups [3, 4, 14, 57]. Data from the Bureau of Labor Statistics of the US Department of Labor (BLS) showed that the incidence of WRUEDs increased from 18% to 65% between 1982 and 1998 [15]. A report in the Netherlands also revealed that there has been increased in WRUED complaints from 19% to 28% between 1997 and 2002, resulting in an annual absence of 8% of the working population due to WRUED [16]. The increased use of personal computers at work is likely associated with an accumulated incidence of WRUEDs [17]. Investigations revealed that the experience of WRUEDs is often pervasive among teaching staff. The annual prevalence of 70% and 46.7% upper extremity disorders were registered among the academic working group in the investigation in Malaysia [5] and Hong Kong [18], respectively. The study in Pakistan also demonstrated that a magnitude of upper extremity disorders was observed in 26.67%, 66.67%, 33.33%, and 53.33% in shoulder, neck, elbow, wrists/hands body regions among the sampled teachers, respectively [19]. Similarly, researchers in Iran [20], Nigeria [21], Egypt [22], and Cameroon [23] documented a prevalence of 42% to 83.5% in the neck, 40% to 62.3% in the shoulder, and a magnitude of 13.9% in wrists/hands. A study in Ethiopia reported pain in the neck (41.5%), and pain in the shoulder (20.5%) [24].

The previous studies documented that WRUEDs have various and interrelated risk factors [25–27]. Age [28], sex [29], work experience [5, 23, 30], and monthly salary [57] are among the sociodemographic factors of WRUEDs. Besides, behavioral/lifestyle, like cigarette smoking, alcohol use, BMI, and physical activities [5, 31] are significant factors. Most conspicuously, extensive investigations have shown that psychosocial factors such as job stress [32–34], job satisfaction [28, 35], and job demand [31] are among the key causes of WRUEDs. Whereas, working hours [36], working posture [30, 37], rest breaks taken, and safety training [38] are among workplace determinants in the manifestation of WRUEDs.

Academicians not only teach students but also engage in activities characterized by prolonged and repetitive work while writing, reading, preparing notes, writing manuscripts for publications, and other activities that have the potential to increase pain intensity and lead to muscle injuries [39], all of which have a significant impact on the development of WRUEDs. However, in developing countries including Ethiopia, it remains uncertain to conclude about the level and conditions giving rise to WRUEDs among academicians in universities. Hence, due to the dearth of up-to-date and reliable figures on upper extremity disorders, it is difficult to establish policies and programs for the prevention and control of such problems. Therefore, determining the prevalence and associated factors of WRUEDs is urgently needed to ensure a sufficient allocation of healthcare resources to address its growing public health problem. It additionally provides data for therapists and allows the affected study subjects to go to therapy treatment to subside the pain and interference of the further episode. The purpose of this study was to explore the prevalence and risk factors of WRUEDs among teaching staff in the University of Gondar, Ethiopia.

## 2. Materials and Methods

### 2.1. Study Design and Period

An institution-based cross-sectional study design was implemented from 17 March to 17 April 2021 to determine the prevalence and explore risk factors influencing WRUEDs among teaching staff in the University of Gondar.

### 2.2. Study Setting and Area

The study was conducted at the University of Gondar. The University of Gondar is found in the oldest and historical place of Gondar City, Northwestern Ethiopia, located 737 km from Addis Ababa, the capital of Ethiopia [40]. The establishment of the University dates back to 1954. Currently, the University has five campuses including the College of Medicine and Health Sciences and Comprehensive Specialized Hospital (CMHS), Maraki, Atse Tewdros, Atse Fasil, and Teda [41]. During the data collection period, there were a total of 2,858 academic staff in all campuses.

### 2.3. Source and Study Populations

All teaching staffs in the University of Gondar were the source population. Whereas, the randomly selected teaching staffs in each campus were the study populations.

### 2.4. Inclusion and Exclusion Criteria

Teaching staff who had at least 1 year of teaching experience and who were available during data collection time were included, while those who were on sick, annual, maternity, and sabbatical leave were excluded. Thus, those who had previous car accidents and injuries were also excluded from the study.

### 2.5. Sample Size Determination and Sampling Procedure

The sample size was calculated using a single population proportion formula [42], with the following assumptions: 5% margin of error (*d*), proportion (*p*) of upper extremity disorders among academicians 50% (no previous study in the study area), 95% confidence interval (CI), and design effect of 1.5 as in the absence of previous literature taking a design effect of 1.5 to 2.0 is suggested [43]. Accordingly, based on a single population proportion formula: *n*=(*Zα*/2)^2^[*p* (1 − *p*)]/*d*^2^; where *n* = initial sample size, *Z* = 1.96, the corresponding Z-score for the 95% CI, *P* = proportion = 50%, *d* = margin of error = 5% = 0.05; and *n*=(1.96)^2^[0.5 (1 − 0.5)]/0.05^2^  = 384.

**Figure 1:**
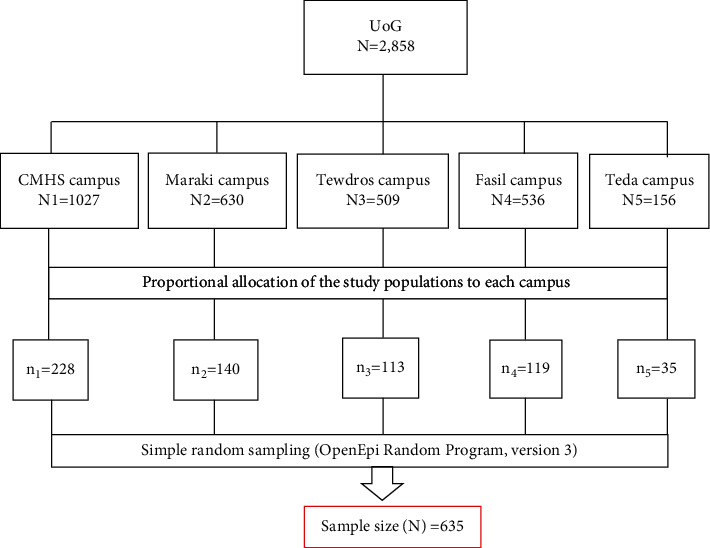
Schematic presentation of sampling procedure for the study of work-related upper extremity disorders among teaching staff in the University of Gondar, Ethiopia.

Using a design effect of 1.5, the calculated sample size with 10% contingency for nonresponse was 635 participants. We employed a stratified sampling technique to select participants from the five campuses of the University of Gondar. The number of sample points was determined by a proportional allocation for each stratum. Hence, there are a total of 1,027 academic staff in College of Medicine and Health Sciences (*N*1 = 1,027), in Maraki campus a total of 630 academic staff (*N*2 = 630), in Tewdros campus a total of 509 academic staff (*N*3 = 509), in Fasil campus a total of 536 academic staff (*N*4 = 536), in Teda campus a total of 156 academic staff (*N*5 = 156). Consequently, the numbers of participants from each campus were 228, 140, 119, 113, and 35 from the College of Medicine and Health Sciences, Maraki, Fasil, Tewodros, and Teda campuses, respectively. Then, the required sample sizes were selected by applying a simple random sampling technique, and OpenEpi random program version 3 was used to randomize academic staff from each stratum (Figure 1).

### 2.6. Operational Definitions

#### 2.6.1. WRUED

Trouble (ache, pain, and discomfort) in upper body site at any time during the last 12 months [57]. The upper body sites include the neck, shoulder, arm/elbow, and wrist/hands.

#### 2.6.2. Repetitive Work

Repeated the same motion for less than 30 seconds with little or no variation for more than 2 hr. Total per day [12].

#### 2.6.3. Body Mass Index

Weight in kilograms divided by the square of the height in meters (kg/m^2^) categorized as underweight = BMI < 18, normal (health) = BMI 18.5–24.9, overweight = BMI 25.0–29.9 = , and obese = BMI ≥ 30.0 [44].

#### 2.6.4. Static Posture

Sitting or standing in a restricted space for two or more hours without changing positions [45].

#### 2.6.5. Cigarette Smoker

Smoking at least one stick of cigarette per day [46].

#### 2.6.6. Alcohol Drinker

The consumption of any kind of alcohol by academic staff at least two times per week [46].

#### 2.6.7. Khat Chewer

Chewing khat three times a week for at least 12 months [47, 48].

#### 2.6.8. Doing Physical Exercise

Doing any kind of sports activity at least two times per week with a duration of at least 30 minutes [49].

#### 2.6.9. Adjustable Chairs

Chairs have wheels or castors suitable for the floor surface have adjustable seat height [50].

#### 2.6.10. Health and Safety Training

Educational actions for credentials by employees, risk factors accountable for musculoskeletal disorders related to work, use of suitable work practices, proper equipment choice, correct use of tools, and adjustments of the workplace [51].

#### 2.6.11. The Habit of Taking Rest Breaks

Every 60 to 120 minutes take a brief rest break. During this break, stand up, move around, and do something else. Get a beverage, take coffee or tea, chat up a coworker, or take a lap around the office [52].

#### 2.6.12. Job Satisfaction

The sum of generic job satisfaction scale score of 32 or above [53].

#### 2.6.13. Job Stress

A workplace stress scale score of 21 or above [54].

### 2.7. Data Collection Tools and Procedures

Data were collected through a standardized self-administered structured questionnaire. The survey questions comprise four sections containing different items. The first section, socio-demographic characteristics assesses information on age, sex, educational status, working experience, and monthly salary. The second category encompasses questions to assess information on upper extremity disorders. A structured self-administered questionnaire adopted from the Nordic musculoskeletal tool (standardized) was used to evaluate upper extremity disorders [55]. The questionnaire has been widely used in previous studies in the Ethiopian context [38, 57]. The third part of the questionnaire includes behavioral factors and psychosocial factors like cigarette smoking (yes/no), BMI (kg/m^2^), physical activity, alcohol consumption (yes/no), history of systemic illness, job satisfaction, and job stress. We used the 10-item generic job satisfaction scale questionnaire to measure academician-perceived job satisfaction [53]. Perceived job-related stress of the participants was collected using the 8-item workplace stress scale questionnaire [54]. The instruments used in this study have been employed in previous studies conducted in the country's context [24, 33, 56]. The fourth part encloses characteristics of the working environment including working hours per day, the habit of taking rest breaks, type of sitting chair, working postures (sitting or standing and repetitive work), ergonomic training, and methods of carrying a laptop. Finally, the self-administered questionnaire was distributed to all eligible participants at their workplaces.

### 2.8. Data Quality Control

The questionnaire was first developed in English and translated into the local language Amharic and back to English by language experts and physiotherapists to ensure its consistency. Three BSc nurses working in the University of Gondar comprehensive specialized hospital were involved in data collection after they took adequate training and orientation. MPH environmental health supervisor working in the College of Medicine and Health Sciences at the University of Gondar was recruited. The data collectors and supervisor had taken orientation on issues relating to the clarity of the questions, objectives of the study, confidentiality of information, and the voluntary involvement (consent) in the study, and on time of data collection as study participant's regular duties should not be compromised. The principal investigator supervised both data collectors and supervisors. To test the validity and reliability of the questionnaire, we conducted a pretest 1 week before the actual data collection on 5% (31) of the sample size at Teda Health Sciences College in Gondar city, and the college was not included in the main survey. Based on the finding from the pretest analysis, a few modifications such as some misinterpretations and ambiguities corrected, and the time taken for the data collection was estimated. In case of any problem during the data collection, the feedback was given by discussing it with the principal investigator, supervisor, and data collectors.

### 2.9. Data Processing and Analysis

Data were checked for completeness and entered into Epi-data version 4.6 and then exported to STATA version 14 for further analysis. We performed descriptive statistics and presented the results with narration, tabulation, and graphical presentation. Normality, outliers, and multicollinearity of the variables were checked before running bivariable and multivariable binary logistic regression analysis where multicollinearity assumption was checked by a variance inflation factor (VIF) and all variables showed values of <5. Thus, we found no evidence of multicollinearity. The reliability of the standardized Nordic Musculoskeletal Questionnaire was tested using Cronbach's alpha, which was found to be 0.7685. According to Cronbach's alpha, the reliability of an instrument is tolerable in a given context at a cutoff point up to 0.65. The 10-item job satisfaction scale questionnaire was also examined for its reliability, and Cronbach's alpha was found to be 0.7874. We also checked the 8-item job stress scale questionnaire, and Cronbach's alpha result was found as 0.824. The instruments were, therefore, tolerable for their consistency in repeating what had previously been measured using these tools.

The association between dependent and independent variables was computed with a binary logistic regression. Variables with *pp* values of <0.2 in the bivariable logistic regression analysis were exported to a multivariable logistic regression to control the potential effects of confounders. Finally, statistically significant variables were established at *p* value <0.05 in a multivariable binary logistic regression model, and an adjusted odds ratio (AOR) with a CI of 95% was reported to measure the strength of association. The final model was checked for goodness-of-fit using the Hosmer–Lemeshow test, and the result explained a good fit (*p*=0.34).

## 3. Results

### 3.1. Socio-Demographic Characteristics of Participants

A total of 607 questionnaires were completed correctly which gave a response rate of 95.60%. From the total participants, 219 academicians (36.08%) from the college of medicine and health sciences, 132 academicians (21.75%) from the Maraki campus, and 107 academicians (17.63%) from the Atse Tewdros campus, 116 academicians (19.11%) from the Atse Fasil campus, and 33 academicians (5.44%) from the Teda campus were selected. More than two-thirds, 76.28% of the participants were males. The participants' age was ranged from 21 to 70 with a mean (±SD) of 32.39 (±6.80) years. The majority of them 362 (59.64%) of the participants indicated they were married. Regarding educational status, 416 (68.53%) of the participants were master's degree holders and 283 (46.62%) of the participants had more than 9 years of working experience (Table 1).

### 3.2. Behavioral and Psychosocial Characteristics

Among the study participants, 108 (17.79%) of them reported they were cigarette smokers. Whereas, 148 (24.38%) stated they had alcohol drinking habits, and 373 (61.45%) of them conveyed they were performing physical exercise at least two times per week. Majority of the respondents, 434 (71.50%) a normal (18.5–24.9 kg/m^2^) body mass index (BMI) and 48 (7.91%) underweight (>18.5 kg/m^2^). Thirty-two (5.27%) participants clarified that they had a history of systemic illness. Regarding psychosocial characteristics, the majority (61.45%) of the academicians demonstrated they were not satisfied with their current job. Regarding job stress, 276 (45.47%) of the respondents stated they perceived stress due to their jobs (Table 2).

### 3.3. Work Environment and Ergonomics Characteristics

One-fifth (20.17%) of the respondents have been worked for more than 8 hours per day. Half (50.74) of the participants explained they had the habit of taking rest breaks at their workplaces. Only 190 (31.30%) of the participants have used adjustable sitting chairs. The majority, 452 (74.46%) of the respondent's jobs involved repetitive movements (activities) and 225 (37.07%) of them did stretching exercises at their workplace. Only, 79 (13.01%) of the respondents reported they took safety training through any kind of media in the past year (Table 3).

### 3.4. Prevalence of WRUEDs

**Figure 2:**
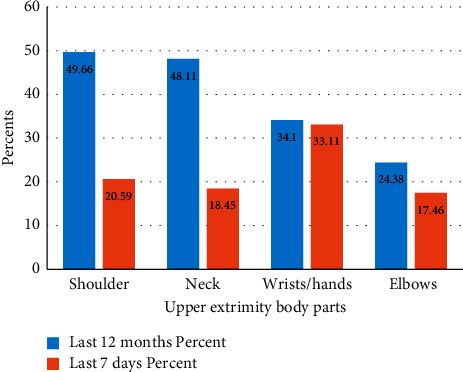
Prevalence of self-reported work-related upper extremity disorders of teaching staff in the University of Gondar, Ethiopia.

The prevalence of WRUED among teaching staff during the last 12 months was 59.14% (*n* = 359) (95% CI (55.1, 63.1)). The most affected body parts were neck pain 292 (48.11%), shoulder pain 301 (49.66%), elbow/forearm pain 148 (24.38%), and hand/wrist pain 207 (34.10%) (Figure 2). There is not a significant difference in prevalence between males and females (45.14% versus 14%), respectively; (*χ*^2^ = 0.001; *p*=0.974) and also there is no significant difference in prevalence between the campuses (21.25%, 12.03%, 11.53%, 10.54%, and 3.79%) in the college of medicine and health sciences, Maraki campus, Atse Tewdros campus, Atse Fasil, and Teda campuses respectively; (*χ*^2^ = 4.8337; *p*=0.305). Near half, 291 (47.94%) of the respondents were prevented from doing their normal work because of pain from 1 to more than 30 days. Furthermore, the length of time they had pain also ranges from 1 to more than 30 days.

### 3.5. Factors Associated with WRUEDs

In the bivariable binary logistic regression analysis, age, marital status, monthly salary, working experience, working hours per day, physical exercise, the habit of taking a break, use of an adjustable sitting chair, and job satisfaction were the factors associated with upper extremity disorders. However, after controlling for confounding variables in the multivariable binary logistic regression analysis, only working hours per day, physical exercise, and job satisfaction remained to have a significant association with upper extremity disorders.

The probability of developing WRUEDs was 2.37 times greater in employees who worked more than 8 hours per day compared to those who worked for 8 hours or less per day [AOR: 2.37; 95% CI (1.40, 4.00)] at a *p*-value of 0.001. Moreover, the odds of having WRUEDs were 2.34 times more likely among workers who did not perform physical activities than among those who perform [AOR: 2.34; 95% CI (1.60, 3.45)] at a *p*-value of 0.000. On top, employees who had dissatisfied with their job were 2.5 times higher at risk of developing WRUEDs compared to those who had job satisfaction counterparts [AOR: 1.5; 95% CI (1.69, 3.68)] at a *p*-value of 0.001 (Table 4).

## 4. Discussion

Upper extremity disorders have been taken into account as the most significant health-threatening problems imposing irreparable economic and social costs [57]. The incidence of such disorders in advanced societies is expanding and requires more attention by relevant authorities [58]. The higher education work environment is characterized by a highly competitive work nature. In developing, nations employee health and safety programs are overlooked, despite the prevailing poor workplace ergonomic and safety arrangements. In Ethiopia, University teaching staff usually handle extracurricular tasks including conducting and preparing research for publication, providing community services, and managing administrative positions beside the regular teaching activities which may exacerbate the experience of upper extremity disorders. Understanding the magnitude and investigating etiologies of the condition plays a paramount role to establish effective prevention and control strategies. This study aimed to examine the prevalence and factors affecting WRUEDs among teaching staff in the University of Gondar, Ethiopia. The prevalence of WRUED in the past 12-months was found to be 59.1% [95% CI (55.1, 63.1)]. In this study, working hours per day, physical activity, and job satisfaction were all factors that influenced upper extremity disorder. However, age, marital status, work experience, monthly salary, and utilization of an adjustable sitting chair were factors not associated with upper extremity disorder in this study.

The result of this study is comparable with findings in Sri Lanka (56.9%) [59], Iran (60%) [60], and Brazil (58%) [61]. The possible justifications for the conformity could be due to working conditions such as workplace ergonomic setups in many developing countries are usually almost similar. Moreover, the nature of tasks in the academic environment including roles related to teaching and research activities usually resemble in every higher academic institution. Participants in those nations might be also obliged to work in a substandard workplace in an unhealthy manner for prolonged periods, and fewer individuals are aware of musculoskeletal disorder safety measures. Furthermore, the participants in those nations may have a low level of health-seeking behavior [62].

On the contrary, the finding of this study had a higher magnitude compared to the studies conducted in Hong Kong (46.7%) [18], Japan (43.10%) [29], Netherlands (54%) [63], and France (50%) [64]. The possible explanation for the difference might be due to variation in the educational system, study setting, workload, limited breaks, high job demands for academic rank, ergonomic design of the work stations provided for the teachers at their institution or social, cultural, and economic differences between Ethiopia and other countries [24, 65, 66]. Another discrepancy might be due to differences in a study period (our study conducted during COVID-19 pandemic) when peoples' movement exceptionally restricted and sedentary lifestyle prevails.

Hence, the prevalence reported in this study was lower than the prevalence reported in studies conducted in Malaysia (70%) [5], Iran (70.58%) [67], and the United Kingdom (65%) [68]. The possible reason for the observed dissimilarity could be due to workplace illness and injury reporting and management procedures might differ across countries. In Ethiopia's workplace, health and safety practices are weak or at an infancy stage. Therefore, participants might be underdiagnosed and underreported of their work-related disorders (WRUEDs) [33, 57], whereas in those compared countries, there might be better work-related disorders reporting and management procedures. Another possible explanation for this disparity might be due to the difference in pain perception of the workers and the level of awareness and openness to the questions.

Our study sample was comprised of more (76.28%) males than females and half (49.59%) of them were younger age groups (30–39 years old). Commonly, most University academic setting is dominated by males and the younger generation. Studies done in Ethiopia [24], Cameroon [23], and Saudi Arabia [69] had similar age and gender distribution, except for Malaysia [5] and Iran [20] studies, which had more females than males. Even though there is no association between gender and WRUEDs, the prevalence of WRUEDs was higher among male staff when compared with females (45.14% vs 14%). This could be due to men academicians being more likely than women to engage in extracurricular activities such as administrative positions and community service activities in addition to their normal teaching duties. So as a result of their extra work activities, working men may be exposed to a variety of workplace risk factors. Another reason for the increased prevalence of WRUEDs among men in this study could be due to a large number of male participants (76.28%) than females (23.72%).

Academic staff who spent more time at work, particularly over 8 hours per day (overtime), significantly reported more WRUEDs in our study. This corresponds to findings of increased WRUEDs among employees who worked more hours per day [24, 30, 32, 70, 71], especially on a computer [72]. Similarly, it has been proposed that long working hours can relatively decrease the time to relieve stress and recuperate from accumulated exhaustion [71], thereby harming the body and triggering WRUEDs. This evidence could be relevant to some academic employees who work long hours per day.

Lack of physical activity was a significant impact on the likelihood of upper-body musculoskeletal diseases in our study. Other previous studies have investigated similar results [5, 24, 57, 73]. The possible suggestion for this finding is that performing physical exercise regularly might promote muscle strength, which helps retain it from getting easily injured on exposure to hazardous conditions. Performing physical exercise prevents muscles from becoming tired by increasing body metabolism, improving oxygen uptake, raising body temperature, and increasing blood flow to tendons, muscles, and ligaments, all of which improve cellular nutrition. More activity over time can strengthen muscles while also increasing endurance [5, 74, 75]. Another plausible study has explained that doing any type of physical exercise three times a week for 20 minutes promotes the reduction of the pain of different body sites, including the upper extremity bodies [72]. Furthermore, another study explored that lack of exercise increases muscle stiffness and decreases their flexibility, making them susceptible to be damaged easily [57]. This indicates practicing physical activity makes muscles strong to resist spasm, stimulates blood vessels to run proper blood circulation that reduces vessel compression, and help to overcome the pain of the disorders.

According to the findings of this study, there was a significant association between job satisfaction and the occurrence of WRUEDs. Our finding was consistent with previous studies conducted in Ethiopia [38], the United Kingdom [76], Greece [77], and Chinese [78]. The plausible reason might be due to workers who were dissatisfied with their working conditions were more likely to acquire work-related stress, which leads to muscle tension, and then exacerbates the development of pain in upper musculoskeletal disorders [79]. Conversely, workers who were satisfied with their job could manage the job demand, control the imbalance in a better way, and minimize the risk of WRUEDs than their job-dissatisfied counterparts. Other possible explanations might be when they work in a situation with high job satisfaction, a high influence over work-related decisions, and get social support, they are less likely to acquire upper extremity disorders than others [80].

Hitherto, studies conducted on musculoskeletal disorders in Ethiopia have rarely considered a significant part of the working population like teaching staff in the universities. This study produces pertinent information on upper extremity disorders and their influencing factors in the context of academicians in the universities in Ethiopia. Thus, plays a vital role to stakeholders and higher officials in that it helps them capable to generate health and safety programs in educational sectors. It also inspires other investigators to further study the relations of a range of workplace factors and the development of various pains of work-related musculoskeletal systems. However, due to the relatively large sample size employed in the study, we have not employed a posture analysis that could address the degree to which those participants have been exposed to workplace ergonomic factors instead we included possible contributing factors that induce upper extremity disorders. Moreover, as the study used a self-report assessment method, recall bias has not been ruled out resulting in underestimation of their disorders. We recommend future studies to account for diverse sectors and to evaluate employees' ergonomic exposures to verify the association with upper extremity impairments.

## 5. Conclusion

This study divulged upper extremity disorders among university teaching staff are omnipresent, according to this study, with more than three-fifth of the academicians suffering from the condition, and it also indicates that males experienced higher proportions of pain than females. Academic environments, particularly universities in Ethiopia, are noted for being competitive working environments, with academicians expected to handle a variety of roles in addition to their regular teaching duties. Even though working arrangements such as ergonomic setups are poorly designed in many universities, academicians linger in working much of their time in sitting, standing, and twisting in awkward postures. In this study, working for more than 8 hours per day, not performing physical exercise and job dissatisfaction were significantly increased the development of WRUEDs. To minimize the scenario, optimizing working hours per day, having a group regular exercise, and proper management of workplace conditions related to job satisfaction are recommended.

## Figures and Tables

**Table 1:** Socio-demographic characteristics of teaching staff in the University of Gondar, Ethiopia, 2021 (*N* = 607).

Variables	Frequency	Percent (%)
Sex		
Male	463	76.28
Female	144	23.72
Age (years)		
21–29	226	37.23
30–39	301	49.59
>40	80	13.18
Marital status		
Single	245	40.36
Married	362	59.64
Educational status		
Bachelor	94	15.49
Master	416	68.53
Ph.D.	97	15.98
Work experience in years		
<9	324	53.38
>9	283	46.62
Monthly salary (ETB)		
<10,000	99	16.31
10,000–13,000	331	54.53
>13,000	177	29.16
Campus		
CMHS	219	36.08
Maraki	132	21.75
Atse Fasil	116	19.11
Atse Tewdros	107	17.63
Teda	33	5.44

**Table 2:** Behavioral and psychosocial characteristics of teaching staff working in the University of Gondar, Ethiopia, 2021 (*N* = 607).

Variables	Frequency	Percent (%)
Cigarette smoker		
Yes	108	17.79
No	499	82.21
Alcohol consumption habit		
Yes	148	24.38
No	459	75.62
Khat chawing behavior		
Yes	134	22.07
No	473	77.93
Physical exercise		
Yes	373	61.45
No	234	38.55
Body mass index (BMI)		
Underweight	48	7.91
Normal	434	71.50
Overweight and obese	125	20.59
Systemic illness		
Yes	32	5.27
No	575	94.73
Job satisfaction		
Satisfied	234	38.55
Not satisfied	373	61.45
Job stress		
Stressed	276	45.47
Not stressed	331	54.53

**Table 3:** Work environment and ergonomics characteristics of teaching staff working in the University of Gondar, Ethiopia, 2021 (*N* = 607).

Variables	Frequency	Percent (%)
Working hours per day		
≤8 hr	483	79.83
>8 hr	122	20.17
Habit of taking breaks		
Yes	308	50.74
No	299	49.26
Adjustable chair		
Yes	190	31.30
No	417	68.70
Prolonged standing		
Yes	196	32.29
No	411	67.71
Prolonged sitting		
Yes	385	63.43
No	222	36.57
Repetitive activity		
Yes	452	74.46
No	155	25.54
Have safety training		
Yes	79	13.01
No	528	86.99
Stretching exercise		
Yes	225	37.07
No	382	62.93

**Table 4:** Predictors of work-related upper extremity disorders among teaching staff in the University of Gondar, Ethiopia, 2021 (*N* = 607).

Variables	WRUEDs		COR (95% CI)	AOR (95% CI)	*p* value
	Yes	No			
Age					
21–29	123	103	1	1	
30–39	182	119	1.28 (0.90–1.82)	1.17 (0.75–1.82)	0.482^*∗*^
>40	54	26	1.74 (1.02–2.97)	1.33 (0.66–2.67)	0.422^*∗*^
Marital status					
Single	135	110	1	1	
Married	224	138	1.32 (0.95–1.84)	1.24 (0.84–1.84)	0.283^*∗*^
Campus					
CMHS	129	90	0.62 (0.28–1.37)	0.53 (0.24–1.21	0.136
Maraki	73	59	0.54 (0.23–1.21)	0.44 (0.19–1.03)	0.101
Atse Fasil	64	52	0.53 (0.23–1.22)	0.50 (0.22–1.16)	0.110
Atse Tewdros	70	37	0.82 (0.35–1.91)	0.73 (0.31–1.71)	0.465
Teda	23	10	1	1	
Work experience in years					
<9	178	146	1	1	
>9	181	102	1.46 (1.05–2.02)	1.28 (0.85–1.92)	0.243^*∗*^
Monthly salary (ETB)					
<10,000	61	38	0.87 (0.52–1.44)	1.00 (0.57–1.95)	0.977
10,000–13,000	183	148	0.67 (0.47–0.97)	0.75 (0.48–1.18)	0.225^*∗*^
>13,000	115	62	1	1	
Working hours per day					
<8 hr	261	222	1	1	
>8 hr	98	24	3.47 (2.15–5.62)	2.37 (1.40–4.00)	0.001^*∗∗*^
Physical exercise					
Yes	175	59	1	1	
No	184	189	3.04 (0.23–0.47)	2.34 (1.60–3.45)	0.000^*∗∗*^
Habit of taking breaks					
Yes	191	117	1	1	
No	168	131	0.78 (0.57–1.09)	0.91 (0.64–1.29)	0.606^*∗*^
Adjustable sitting chair					
Yes	121	69	1	1	
No	238	179	0.78 (0.53–1.08)	0.80 (0.54–1.18)	0.261^*∗*^
Job satisfaction					
Satisfied	177	57	1	1	
Not satisfied	182	191	3.26 (2.27–4.68)	2.50 (1.69–3.68)	0.001^*∗∗*^

Keys. 1 = reference category, AOR = adjusted odds ratio, CI = confidence interval, COR = crudes odds ratio, CMHS = collage of medicine and health sciences, ^*∗*^ = Significant at a *p* value < 0.2 in bivariable logistic regression analysis, ^*∗∗*^ = significant at a *p* value <0.05 in multivariable logistic regression analysis, WRUEDs = work-related upper extremity disorders, Hosmer and Lemeshow test *p*=0.34.

## Data Availability

The data sets generated and/or analyzed during this study are not publicly available because the data contain indirect identifying characteristics (e.g., age and sex) but are available from the corresponding author on reasonable request (amensisahailu@gmail.com).

## Abbreviations

AOR: Adjusted odds ratio
BMI: Body mass index
BSc: Bachelor of science
CI: Confidence interval
COR: Crude odds ratio
ETB: Ethiopian birr
IF: Variance inflation factor
IPH: Institute of public health
MPH: Master of public health
MSDS: Musculoskeletal disorders
OR: Odds ratio
OSHA: Occupational safety and health administration
UK: United Kingdom
UoG: University of Gondar
WRUEDs: Work-related upper extremity disorders.
